# Parental Imitations and Expansions of Child Language Predict Later Language Outcomes of Autistic Preschoolers

**DOI:** 10.1007/s10803-022-05706-9

**Published:** 2022-08-17

**Authors:** Jodie Smith, Rhylee Sulek, Kailia Van Der Wert, Olivia Cincotta-Lee, Cherie C. Green, Catherine A. Bent, Lacey Chetcuti, Kristelle Hudry

**Affiliations:** 1 School of Allied Health, Human Services and Sport, La Trobe University, Plenty Road and Kingsbury Drive Bundoora, Melbourne, 3086 Australia; 2 Department of Psychology, Counselling and Therapy, La Trobe University, Plenty Road and Kingsbury Drive Bundoora, Melbourne, 3086 Australia; 3 School of Allied Health Sciences, Griffith University, Parklands Drive, Southport, QLD 4222 Australia; 4 School of Psychology, Deakin University, 221 Burwood Highway, Burwood, VIC 3125 Australia

**Keywords:** Language development, Parent–child interaction, Responsiveness, Preschoolers, Verbal imitations, Expansions

## Abstract

Both the amount *and* responsiveness of adult language input contribute to the language development of autistic and non-autistic children. From parent–child interaction footage, we measured the amount of adult language input, overall parent responsiveness, and six discrete parent responsive behaviours (*imitations*, *expansions*, *open-ended questions*, *yes/no questions*, *comments* and *acknowledgements*) to explore which types of responsiveness predicted autistic preschoolers’ language five months later, after controlling for adult language input. We found *expansions* and particularly *imitations* to be more important for later language than overall responsiveness. This study emphasises the need to capture what exactly about parent language input influences child language acquisition, and adds to the evidence that imitating and expanding early language might be particularly beneficial for autistic preschoolers.

Acquisition of oral language is an important developmental milestone. In non-autistic cohorts, language is not only fundamental for communicating with others but a prerequisite for the advancement of other skills such as emergent literacy (Zubrick et al., 2015), attention and prosocial behaviour (Bretherton et al., 2013). There is likewise growing evidence that early language skills are predictive of social, academic, adaptive, and vocational outcomes in autistic children (Durkin et al., 2011; Gillespie-Lynch et al., 2012; Howlin et al., 2000). Building awareness of specific factors that robustly contribute to early language acquisition is important if we are to optimise developmental outcomes for children on the autism spectrum.

## Adult–Child Interaction and Language Development

Language learning takes place in social contexts beginning with interactions between parents and infants (Bruner, 1975; Vygotsky, 1962). Studies of the general population highlight that both the quantity (i.e., overall amount) and quality (i.e., syntactic and semantic diversity) of adult language input during early interactions shape children’s early language trajectories (Rowe, 2012; Smith et al., 2021). There is likewise compelling evidence of similar associations for parental input supporting the language acquisition of autistic children (Naigles, 2013). As an illustration, Fusaroli et al. (2019) discovered that autistic preschoolers had longer utterances, together with larger, richer vocabularies, when they had been exposed to longer parent utterances earlier in childhood. Swanson et al. (2019) similarly found that exposure to more adult words at ages 9 and 15 months was associated with better subsequent language at age 24 months for both infants at higher- and lower-likelihood of developing autism.

Another unique predictor of child language outcomes in non-autistic cohorts is adult responsiveness (Eshel et al., 2006)—that is, the extent to which adults provide responses that are timely, semantically contingent, and developmentally appropriate in relation to what a child has said or done (Tamis-LeMonda et al., 2001). As autistic children can have difficulties with joint attention and shifting social attention (Adamson et al., 2019; Mo et al., 2019), adult responsiveness may be particularly important for facilitating language for young autistic children (Hudry et al., 2013; McDuffie & Yoder, 2010; Siller & Sigman, 2002) since reduced attentional demands should theoretically increase their capacity for word learning (Hoff, 2006). Notably, since quality and quantity language measures are largely consistent across the literature and easily replicable—i.e., often operationalised as amount/diversity of adult words and mean length of utterances (MLU)—comparing findings here is relatively straightforward. In contrast, cross-study comparison of findings in relation to adult responsiveness is complicated by opaque and inconsistent measurement.

## Global vs. Discrete Measurement of Parent Responsiveness

Parent responsiveness is typically operationalised through coding parent–child interaction footage using measures that are either global (i.e., rating/Likert scales) or discrete (i.e., capturing “moment-to-moment” responsiveness) (Barnett et al., 2022; Morawska et al., 2014). In a recent systematic review and meta-analysis, Edmunds et al. (2019) found a significant positive effect of parent responsiveness on communication outcomes for children with, or at higher likelihood of, autism. This relationship did not differ according to the use of global versus discrete measurement, although there was more variability when global ratings were used. This indicates that while both measurement types can meaningfully capture parent responsiveness, discrete coding may offer a more nuanced picture. Moreover, from an application perspective, global responsiveness measures are often subjective impacting measurement precision (Morawska et al., 2014). Further, global measures comprise multiple behaviours making it difficult to identify which specific aspects of the interaction are facilitating language acquisition (Hirsh-Pasek et al., 2015).

Prior attempts to capture ‘moment-to-moment’ micro-level responsiveness have lacked specificity and/or informative detail; in particular, the coding of individual parent responsive utterances but failure to further subcategorise (i.e., whether a parent’s response is in the form of an imitation, a question or a comment). Such lack of specificity could partly explain inconsistencies—and indeed null findings—in the extant literature here. For instance, Flippin and Watson (2015) measured composite responsive parent verbal utterances (including comments, requests, and directives) provided by mothers and fathers of young autistic children. They found that fathers’ verbal responsiveness was significantly related to overall child language skills, while mothers’ verbal responsiveness was unrelated to child language. Choi et al. (2020) similarly totaled contingent verbal responses (i.e., occurrences of verbal and non-verbal infant communicative behaviours that were followed by a parent response) in dyads with and without a family history of autism, along with parents’ MLU. Whilst MLU was associated with subsequent child language abilities, there was a surprising *null* effect for contingent responses in their study.

## Discrete Responsive Behaviours

Studies in the general population literature that have further categorised discrete parental responsive behaviours into more specific subcomponents (e.g., imitations and expansions of child language, responsive comments etc.) have yielded more consistent findings than those that have relied on composite measures. For example, an association between parent use of both responsive imitations (i.e., Child: “Car”; Adult: “Car”) and expansions (i.e., Child: “Car”; Adult: “Red car”), and concurrent and subsequent child language is now accepted as fairly robust (Levickis et al., 2014; Smith et al., 2018a, 2018b; Tamis-LeMonda et al., 2001). In the context of early childhood autism, however, few studies have taken such a nuanced view of parent responsiveness and none have compared the relative predictive value of overall and discrete parent responsiveness; yet, gathering a more nuanced, fine-tuned understanding of adult responsiveness during interactions with young autistic children may be especially important here. There are likely subtle perturbations—whether from inherent child social communication difficulties or attendant parent behaviours—influencing parent–child interactions with young autistic children (Wan et al., 2013). Consequently, there may be unforeseen generalisability issues with using previously published checklists and measures developed in non-autistic populations within neurodiverse samples.

Of all discrete responsive behaviours, we might expect that behaviours that include copying back child language (i.e., *imitations* and *expansions*) may be more valuable for autistic children than is the case in neurotypical development (Field, 2017). This hypothesis is based on evidence suggesting that being imitated can support increased social behaviours for autistic children, including increased vocalisations (Field et al., 2001), imitations (Field et al., 2008) and communicative gaze (Sanefuji & Ohgami, 2011). These responsive parent behaviours that explicitly echo and/or extend on what a child has said (i.e., imitations and expansions) also shape and encourage early words by promptly reinforcing the accurate adult target word (Tamis-LeMonda et al., 2001) and support communicative intent by providing immediate feedback to the child that verbal sounds have meaning (Bruner, 1975; Rowland & Fried-Oken, 2010). As for other responsive behaviours—such as responsive questions (i.e., Child: Holds a car; Parent: “What’s that?” [WH question] or “Is that a car?” [yes/no question]) and comments/acknowledgements (i.e., Child: Holds a car; Parent: “That’s a car!” [comment] or “Wow!” [acknowledgement])—it is plausible that these may have less influence on early language acquisition because they do not immediately reflect back or build upon a child’s own words/sounds. This may be especially true for autistic children who may benefit most from the strengthening of intentional communication afforded by immediacy and attentional contingency.

There is less consistency in the literature with regard to the effect of parent responsive behaviours on children’s language skills in the context of autism, compared to in the general population. For example, *expansions* have sometimes been related to concurrent and subsequent child language (McDuffie & Yoder, 2010; Naigles, 2013) and sometimes not (Haebig et al., 2013). Surprisingly, *imitations* have been found to be unrelated to later language in two previous studies of parents and young autistic children (Haebig et al., 2013; McDuffie & Yoder, 2010). And, responsive *commenting* has been found to predict change in child expressive vocabulary in one study (McDuffie & Yoder, 2010) but was unrelated to later language for more verbally fluent children in a subsequent study (Haebig et al., 2013). We know little about the role of responsive *questions* and *acknowledgements* on later language outcomes in autistic cohorts. Small samples sizes and few dedicated studies may contribute to the inconsistency we see in the autistic literature versus that on non-autistic children, necessitating replication studies and larger cohorts.

## Current Study

Drawing on data from a cohort of parents and their autistic preschoolers, we sought to investigate the relative predictive value of six discrete parent responsive behaviours for later child language. We broadly hypothesised that:

1. Discrete responsive behaviours would account for more variance in receptive and expressive child language outcomes than overall parent responsiveness, and;
2. The six discrete behaviours would contribute differentially, with behaviours that explicitly echo and/or extend on what a child has said (i.e., *expansions* and *imitations*) accounting for more variance than those that do not (i.e., *WH questions*, *yes/no questions*, *comments* and *acknowledgements)*.

## Method

### Design and Procedure

This study utilised data collected from a larger longitudinal study of autistic children and their families conducted between 2017 and 2019. Ethics approval for the study was obtained from La Trobe University Human Research Ethics Committee; HREC #16-136. Children were eligible for the study if aged 17–43 months, diagnosed with autism and with a non-verbal developmental age-equivalence ≥ 12 months. Parents also needed to speak sufficient English to understand written and spoken information to partake in this research. Autism diagnoses were confirmed through administration of the Autism Diagnostic Observation Schedule (ADOS-2; Lord et al., 2012) by a research-reliable assessor. Measures of child developmental skills (including language) and parent–child interaction (PCI) footage were collected during research assessments completed at baseline and five-month follow-up. Where two parents attended assessments, the self-designated primary caregiver participated in the PCI video.

### Participants

Table 1 provides baseline characteristics for the 53 parent–child dyads comprised in this study. Most children were male with an average age under three years. Mean verbal and non-verbal developmental skills were below chronological age expectations. Participating parents were largely well educated, biological mothers. A substantial minority subgroup of parents reported low-income status (*n* = 3 missing), and 24 families self-described as culturally and linguistically diverse (with varied home languages including Mandarin, Cantonese, Tamil and others). Codes for baseline parent–child interaction were available for 49 parents (*n* = 4 missing; reasons for missing videos included declined consent to be filmed and predominant use of a language other than English in videos).

**Table 1:** Baseline characteristics of participating parents and children

Characteristics	*N*	*M*/*n* (*SD*/%)	Range
Child			
Age (months)	53	33.87 (6.92)	17–43
Sex (male)	53	43 (81.13)	
Mullen Scales of Early Learning Developmental Quotient	53		
Non-verbal		71.56 (23.55)	31–133
Verbal		58.20 (32.70)	12–134
MacArthur–Bates communicative development			
Receptive vocabulary	43	245.05 (179.41)	0–669
Expressive vocabulary	43	141.49 (184.49)	0–630
Autism diagnostic observation schedule CSS	53	7.06 (1.68)	3–10
Primary parent			
Mothers	53	45 (84.91)	
Age (years)	45	36 (4.56)	26–48
Education level	42		
Primary		1 (2.38)	
Secondary		6 (14.29)	
Tertiary		19 (45.24)	
Postgraduate		16 (38.10)	
Family			
Culturally and linguistically diverse	45	24 (53.33)	
Low income status	45	16 (35.56)	
Primary home languages other than English (top 6)			
Mandarin	47	6 (12.77)	
Tamil	47	4 (8.51)	
Japanese	47	1 (2.13)	
Urdu	47	1 (2.13)	
Vietnamese	47	1 (2.13)	
Sinhala	47	1 (2.13)	

### Measures

#### Predictors: Parent Responsive Behaviours

Parent–child interaction sampling was in the context of ten minutes of free-play with an identical set of age-appropriate toys provided to each parent (i.e., books, blocks, pretend play materials, a spinning top etc.). Videos were transcribed and coded using Systematic Analysis of Language Transcripts (SALT) software following pre-prescribed coding conventions (Miller et al., 2011). Basic SALT measures for both parent and child (i.e., total words and MLU) were retained for inclusion as potential covariates in the current analysis (see below). Videos and transcripts were then reviewed and all parent utterances that were timely (within < 5 s following a child behaviour or utterance), semantically contingent, and developmentally appropriate (Tamis-LeMonda et al., 2001) were coded as *responsive*. *Proportionate responsiveness* was computed for analysis as the number of responsive utterances divided by total number of parent utterances. Videos and transcripts were reviewed again to further sub-code all responsive utterances into exhaustive and mutually-exclusive categories of *imitations*, *expansions*, *WH questions*, *yes/no questions*, *comments or acknowledgements.* Table 2 presents detailed descriptions and summary descriptive statistics of responsive behaviours. Coded procedures were based on past research (see Levickis et al., 2014; Smith et al., 2019).

**Table 2:** Detailed descriptions of baseline parental responsiveness measures (predictors) and follow-up child language (outcomes)

Variable	Type	Definition	Sample (*n*)	M (SD), range
Predictor	Responsiveness (proportionate)	Proportion of parent utterances that were responsive to child language or behaviour. Calculated by dividing responsive utterances by overall number of utterances	49	.48 (.17), .11–.82
Predictor	Imitations	Parent repeated the child’s preceding verbal or non-verbal vocalisation or verbalisation exactly or with a reduction of words (e.g., Child: “*Car*”; Parent: “*Car*”)	49	5.82 (7.20), 0–35
Predictor	Expansions	Parent repeated one or all of the child’s preceding words/sounds and added language (e.g., Child: “*Car*”; Parent: “*It is a car!*”)	49	4.04 (5.23), 0–19
Predictor	WH questions^a^	Parent asked a “wh” question (e.g., “what,” “when,” “who”), following a child act or behaviour (e.g., Child picks up a car; Parent: “*What’s that?”*). ‘How’ questions included here too	49	4.27 (4.19), 0–23
Predictor	Yes/No questions^a^	Parent asked a question requiring a binary (yes or no) answer following a child act or behaviour (e.g., Child picks up a car; Parent: *“Is that a car?”*)	49	7.45 (7.04), 0–28
Predictor	Comments^a^	Parent commented on a child’s action or behaviour (e.g., Child plays with a car; Parent: “*That’s a car!”*). No response required from the child. Excluded acknowledgements/exclamations	49	45.27 (22.31), 5–91
Predictor	Acknowledgements^a^	Exclamation or acknowledgement of a child’s action or behaviour (e.g., Child crashed the car; Parent: “*Uh-oh!”*)^b^. No response required from the child	49	22.20 (12.86), 3–61
Outcome	Mullen Scales of Early Learning (MSEL)	Examiner assessed measure of child development. Expressive and Receptive Language Subscales retained with age-equivalent scores (in months) used for analysis	40	Expressive^c^: 24.83 (12.87), 3–45 Receptive^c^: 25.17 (14.34). 2–55
Outcome	Vineland Adaptive Behavior Scales (VABS)	Parent-report measure of child adaptive functioning collected via parent interview. Expressive and Receptive Language Subscales retained with age-equivalent scores (in months) used for analysis	50	Expressive^c^: 25.80 (12.73), 5–60 Receptive^c^: 26.12 (14.67). 3–66
Outcome	MacArthur-Bates communicative development (MCDI)	Parent-report measure of the number of words a child understands and uses collected via parent questionnaire. Cumulative expressive and receptive vocabulary scores used for analysis	44	Expressive: 257.75 (235.66), 0–669 Receptive: 369.91(194.89). 0–675

^a^As coding was mutually exclusive, if parent utterance also imitated or expanded child utterance, imitation/expansion took precedence; ^b^common responses included: *yeah*, *no*, *wee*, *wow*, *oh-dear*, *mm*, *okay*, *hooray*; ^c^ age-equivalence scores (in months)

Interrater reliability was evaluated by having 20% of randomly-selected videos coded by a second trained researcher. We follow Syed and Nelson (2015)’s recommendation of reporting multiple indices of reliability; here, Intraclass Correlation Coefficients (ICC) and Cohen’s kappa statistics. Reliability was good-to-excellent for *proportionate responsiveness* (κ = .79, ICC > .99), *imitations* (κ = .70, ICC = .93), *expansions* (κ = .65, ICC = .97), *WH questions* (κ = 1.00, ICC = 1.00), *comments* (κ = .75, ICC = 1.00) and *acknowledgements* (κ = .69, ICC = .98), and acceptable for *yes/no questions* (κ = .56, ICC = .97).

#### Outcomes: Child Language

Available standardised measures of child language included Expressive and Receptive subscales of the Mullen Scales of Early Learning (MSEL; Mullen, 1995) and Vineland Adaptive Behavior Scales 2nd Edition (VABS-II; Sparrow et al., 2005), as well as vocabulary data from the MacArthur-Bates Communicative Development Inventories (MCDI; Fenson et al., 2007). The MSEL Expressive and Receptive Language subscales have acceptable internal consistency (*α* > .85) and good construct validity against other measures of cognitive ability (*r* = .74) (Swineford et al., 2015). The VABS-II has good inter-interviewer reliability for the Communication Domain (*r* = .76) and has been validated for use with autistic children (Sparrow et al., 2005). The MCDI also has good reliability and predictive validity for later language in autistic children (Luyster et al., 2007). To avoid floor effects in our data, we used MSEL and VABS-II Expressive and Receptive subscale age equivalent scores in our analysis. For MCDI data, we retained raw counts of expressive and receptive vocabulary.

#### Covariates

We considered several child and parent factors as potential covariates for inclusion in analyses. Due to the small sample size, a preliminary correlation analysis was conducted to determine the associations between child language outcomes and predictor measures coded from PCI footage, and each of child autism severity (ADOS-2 calibrated severity scores, range 1–10; Lord et al., 2012), cognitive ability (MSEL Developmental Quotient [DQ]; Mullen, 1995) and adaptive behavior (Adaptive Behavior Composite [ABC]; Sparrow et al., 2005). Our SALT measures of child language from interaction videos (i.e., linguistic opportunities for parent responsiveness)—namely child total words (CTW), child different words (CDW) and child mean length of utterance (MLU)—were highly intercorrelated (*r* = .686 to .850). For parsimony, of all child measures, we only retained CTW (*M* = 57.73, SD = 71.76, Range: 0–256) as a covariate because it showed the strongest associations with other child measures: cognitive ability (*r* = .686) and adaptive behaviour (*r* = .531). Parent MLU (*M* = 2.78 SD = 87, Range: 1.34–5.01) was included as a second covariate to control for the quality of parent linguistic input (Fusaroli et al., 2019; Swanson et al., 2019).

#### Data Analysis

We note that study data collection coincided with local community lockdowns related to COVID-19, resulting in a loss of MSEL (i.e., direct assessment) data for 24.5% (*n* = 13) of the sample. Across the remaining variables of interest, missing data ranged between 5.7 and 17%, and were at random χ^2^(24) = 34.80, *p* = .71. All analyses have been conducted with available data only.

A series of Pearson’s correlations were run to identify relationships between our measures. Significant correlates were then entered into a series of hierarchical multiple regressions to determine the additional predictive value of parent responsiveness (entered at Step 2; either proportionate parent responsiveness *or* discrete parent responsiveness behaviours) for child receptive and expressive language outcomes, beyond the effect of covariates (entered at Step 1; CTW, parent MLU and home language). Two PCI videos were partly in a language other than English. A sensitivity analysis conducted to see if the final results changed when these were included/excluded yielded substantively identical results, so data are reported for all coded videos (*n* = 49).

## Results

Table 3 shows a pattern of moderate-to-strong significant correlations between all child language outcome measures and the covariates parent MLU and CTW. *Proportionate responsiveness* was moderately strongly correlated with MSEL receptive language (*r* = .439) and weakly so with VABS-II expressive language (*r* = .439). All child language outcomes were moderately correlated with *imitations* (*r* = .315–555), and moderate-to strongly correlated with *expansions* (*r* = .421–.799). Significant, weak-to-moderate correlations were found between *WH questions* and VABS-II receptive (*r* = .378) and expressive language (*r* = .451) and MCDI expressive vocabulary (*r* = .309), with a weak correlation found between *yes/no questions* and MSEL expressive language (*r* = .340).

**Table 3:** Full correlation matrix of covariates, predictors and outcomes

	PMLU	CTW	ACK	COM	YN	WH	IMI	EXP	Prop Resp	MSEL RL	MSEL EL	VABS RL	VABS EL	MCDI RL	MCDI EL
Income	.452**	.224	.278	.219	.100	.204	.125	.295	.073	.127	.142	.259	.373*	.291	.323*
*n*	42	42	42	42	42	42	42	42	42	34	34	43	43	40	40
PMLU		.652**	.170	.279	.235	.237	.135	.585**	.164	.578**	.674**	.482**	.703**	.743**	.694**
*n*		49	49	49	49	49	49	49	49	38	38	47	47	41	41
CTW			− .094	− .109	.187	.304*	.319*	.798**	.178	.748**	.725**	.463**	.721**	.744**	.782**
*n*			49	49	49	49	49	49	49	38	38	47	47	41	41
ACK				.589**	.479**	.476**	− .101	.058	.480**	− .046	− .077	.054	.060	− .163	− .081
*n*				49	49	49	49	49	49	38	38	47	47	41	41
COM					.544**	.277	.292*	.140	.618**	.211	.126	.149	.134	− .020	− .024
*n*					49	49	49	49	49	38	38	47	47	41	41
YN						.643**	.283*	.334*	.626**	.292	.340*	.259	.268	.124	.198
*n*						49	49	49	49	38	38	47	47	41	41
WH							.207	.508**	.595**	.201	.315	.378**	.451**	.206	.309*
*n*							49	49	49	38	38	47	47	41	41
IMI								.484**	.315*	.447**	.407*	.555**	.470**	.395*	.405**
*n*								49	49	38	38	47	47	41	41
EXP									.421**	.732**	.635**	.598**	.799**	.688**	.724**
*n*									49	38	38	47	47	41	41
Prop Resp.										.439**	.285	.200	.303*	.197	.246
*n*										38	38	47	47	41	41
MSEL RL											.831**	.551**	.780**	.813**	.827**
*n*											40	39	39	36	36
MSEL EL												.585**	.854**	.841**	.911**
*n*												39	39	36	36
VABS RL													.814**	.698**	.659**
*n*													50	44	44
VABS EL														.857**	.887**
*n*														44	44
MCDI RL															.926**
*n*															44

*PMLU* parent mean length of utterance, *CTW* child total words, *ACK* acknowledgments, *COM* comments, *YN* yes/no questions, *WH* WH questions, *IMI* imitations, *EXP* expansions, *Prop Resp* proportionate responsiveness, *MSEL RL* Mullen Scales of Early Learning receptive language age equivalence, *MSEL EL* Mullen Scales of Early Learning expressive language age equivalence, *VABS RL* Vineland Adaptive Behaviour Scales receptive language age equivalence, *VABS EL *Vineland Adaptive Behaviour Scales expressive language age equivalence, *MCDI RL* MacArthur-Bates communicative development inventories receptive vocabulary, *MCDI EL* MacArthur-Bates communicative development inventories expressive vocabulary

*p < .05, **p < .001

### Prediction of Child Language Outcomes

Tables 4, 5, and 6 present results of hierarchical multiple regressions. Where both types of responsiveness (i.e., *proportionate* and discrete behaviours) were related to child language, the predictive value of each of these for a given outcome measure was evaluated in separate regression models (as these were non-independent so could not be included within the same model).

**Table 4:** Hierarchical multiple regression for directly assessed expressive and receptive language measures (MSEL)

Receptive language						Expressive language					
	*β*	*R*^2^	*F*	*ΔR*^2^	*ΔF*		*β*	*R*^2^	*F*	*ΔR*^2^	*ΔF*
Model 1											
Step 1		.60	14.38**	–	–						
PMLU	.205										
CTW	.648**										
Home language	.086										
Step 2		.69	15.90**	.10	8.83*						
PMLU	.130										
CTW	.658**										
Home language	.107										
Proportionate responsiveness	.321*										
Model 2											
Step 1		.60	14.38**	–	–	Step 1		.64	17.14**	–	–
PMLU	.205					PMLU	.370*				
CTW	.648**					CTW	.514**				
Home language	.086					Home language	− .001				
Step 2		.68	11.51**	.08	3.49*	Step 2		.68	9.38**	.05	1.22
PMLU	.143					PMLU	.349*				
CTW	.432*					CTW	.413*				
Home language	.023					Home language	− .042				
Imitations	.059					Imitations	.172				
Expansions	.344					Expansions	.076				
						Yes/No Qs	.042				

*p < .05, **p < .001

**Table 5:** Hierarchical multiple regression for parent reported expressive and receptive language measures (VABS)

Receptive language						Expressive language					
	*β*	*R*^2^	*F*	*ΔR*^2^	*ΔF*		*β*	*R*^2^	*F*	*ΔR*^2^	*ΔF*
Model 1						Step 1		.57	16.45**	–	–
						PMLU	.425*				
						CTW	.418*				
						Home Language	.020				
						Step 2		.60	13.65**	.03	2.82
						PMLU	.420*				
						CTW	.416*				
						Home Language	.077				
						Proportionate Responsiveness	.186				
Model 2											
Step 1		.27	4.46*	–	–	Step 1		.57	16.45**	–	–
PMLU	.398*					PMLU	.425*				
CTW	.201					CTW	.418*				
Home language	.199					Home Language	.020				
Step 2		.52	6.13**	.25	6.00*	Step 2		.76	17.92*	.19	8.89**
PMLU	.404*					PMLU	.394*				
CTW	− .114					CTW	.086				
Home language	.113					Home Language	− .036				
Imitations	.406*					Imitations	.225*				
Expansions	.191					Expansions	.335*				
‘Wh’ Questions	.137					‘Wh’ Questions	.123				

*p < .05, **p < .001

**Table 6:** Hierarchical multiple regression for parent reported expressive and receptive vocabulary (MCDI)

Receptive vocabulary						Expressive vocabulary					
	*β*	*R*^2^	*F*	*ΔR*^2^	*ΔF*		*β*	*R*^2^	*F*	*ΔR*^2^	*ΔF*
Step 1		.66	21.50**	–	–	Step 1		.66	22.04**	–	–
PMLU	.469*					PMLU	.346*				
CTW	.425*					CTW	.555**				
Home language	.030					Home language	.063				
Step 2		.74	17.84**	.08	4.92*	Step 2		.73	14.42**	.08	2.97*
PMLU	.510**					PMLU	.384*				
CTW	.244*					CTW	.366*				
Home language	− .048					Home language	.004				
Imitations	.281*					Imitations	.237*				
Expansions	.084					Expansions	.098				
						‘Wh’ Questions	.062				

*p < .05, **p < .001

### Predictors of Assessed Child Language

In the prediction of child MSEL receptive language, covariates entered at Step 1 (CTW, parent MLU and home language) contributed significantly to the model, explaining 59.8% of the variance, although only CTW made a significant unique contribution (β = .658, *p* < .001). The addition of *proportionate responsiveness* at Step 2 added significant predictive value, explaining a further 9.6% of the variance, and with both *proportionate responsiveness* (β = .321, *p* = .006) and the covariate CTW (β = .658, *p* < .001) significant unique predictors of MSEL receptive language; R^2^ = .69, F(4, 28) = 15.90, p < .001. In the alternate model, with discrete responsiveness behaviours (specifically, *imitations* and *expansions)* instead added at Step 2, these explained a significant further 8.3% of variance in scores; R^2^ = .68, F(5, 27) = 11.51, p < .001. Here, however, only the covariate CTW (β = .432, *p* = .013), but neither discrete predictor, *imitations* (β = .059, *p* = ns) or *expansions* (β = .344, *p* = ns), was a significant unique predictor.

In the prediction of MSEL expressive language, covariates (CTW, parent MLU and home language) entered at Step 1 again contributed significantly to the model, explaining 61.1% of the variance, with both CTW (β = .514, *p* = .001) and MLU (β = .370, *p* = .020) showing unique contributions. The addition of discrete responsiveness behaviours (here, *imitations*, *expansions* and *yes/no questions*) at Step 2 explained a further (but non-significant) 4.5% of the variance in MSEL expressive language scores, with covariates CTW (β = .413, *p* = .023) and parent MLU (β = .349, *p* = .032) as significant, unique predictors in the final model; R^2^ = .68, F(6, 26) = 9.38, p < .001. As *proportionate responsiveness* was not found to be associated with MSEL expressive language in preliminary correlation analyses, this model was not tested.

### Predictors of Parent-Reported Child Communication Skills

In the prediction of VABS receptive communication, covariates, CTW, parent MLU and home language entered at Step 1 explained 27.2% of the variance, with only MLU conferring a significant unique contribution (β = .398, *p* = .045). The addition of *discrete* responsiveness behaviours (here, *imitations*, *expansions* and *WH questions*) at Step 2 explained a further 25.4% of the variance in VABS receptive communication, with *imitations* (β = .406, *p* = .007), but not *expansions* (β = .191, *p* = ns) or *WH questions* (β = .137, *p* = ns), emerging as a significant unique predictor, along with one covariate, parent MLU (β = .404, *p* = .021); R^2^ = .52, F(6, 34) = 6.13, p < .001. No model was tested with *proportionate parent responsiveness* entered at Step 2.

In the prediction of VABS-II expressive communication, the combination of covariates, CTW, parent MLU and home language, entered at Step 1 explained 57.1% of the variance, with CTW (β = .418, *p* = .005) and MLU (β = .425, *p* = .006) making a significant unique contribution*.* The addition of *proportionate responsiveness* at Step 2 explained a further, non-significant, 3.1% of the variance in VABS-II expressive communication, with both covariates remaining as significant, unique predictors with similar coefficients; R^2^ = .60, F(4, 36) = 13.65, p < .001. In the alternative model with the addition of discrete behaviours (here, *imitations*, *expansions* and *WH questions*) at Step 2, these explained a further significant 18.8% of the variance in VABS-II expressive communication scores; R^2^ = .76, F(6, 34) = 17.92, p < .001. Here, both *imitations* (β = .225, *p* = .031) and *expansions* (β = .335, *p* = .036) carried significant unique predictive value, along with one covariate, parent MLU (β = .394, *p* = .002).

### Predictors of Parent-Reported Vocabulary Knowledge

In the prediction of MCDI receptive vocabulary, the combination of covariates (CTW, parent MLU and home language) entered at Step 1 explained 65.5% of the variance, with CTW (β = .425, *p* = .004) and MLU (β = .469, *p* = .002) making a significant unique contribution. The addition of discrete responsiveness behaviours (here, *imitations* and *expansions*) at Step 2 explained a further, significant 8.1% of the variance in MCDI receptive vocabulary language, although only *imitations* was a significant unique predictor (β = .281, *p* = .016), alongside two covariates, parent MLU and CTW; R^2^ = .74, F(5, 32) = 17.84, p < .001. No model was tested with *proportionate parent responsiveness* entered at Step 2.

Finally, in the prediction of MCDI expressive vocabulary, the combination of covariates (CTW, parent MLU and home language) entered at Step 1 explained 66.0% of the variance, with CTW (β = .555, *p* < .001) and MLU (β = .346, *p* = .019) making a significant unique contribution. The addition of discrete responsiveness behaviours (here, *imitations*, *expansions and WH questions*) at Step 2 explained a further 7.6% of the variance in scores, although again, only *imitations* (β = .237, *p* = .042) carried significant unique predictive value, alongside two covariates, parent MLU and CTW; R^2^ = .74, F(6, 31) = 14.42, p < .001. No model was tested with *proportionate parent responsiveness* entered at Step 2.

## Discussion

This study took a more nuanced approach to exploring the contribution of parent responsiveness to child language outcomes compared to previous work in the autism literature. Building upon previous research, we considered the comparative contribution of six discrete responsive behaviours (*expansions*, *imitations*, *WH questions*, *yes/no questions*, *comments* and *acknowledgements*) and parents’ relative responsiveness to later child language in a diverse sample of parents and their autistic preschoolers. As hypothesised, relative responsiveness was less predictive of later language skills than discrete responsive behaviours, predicting only one language outcome (i.e., directly-assessed receptive language). Also, as hypothesised, expansions—but especially imitations—of child language were valuable for later language. Whereas responsive questions, comments and acknowledgments did not predict any language outcomes, imitations contributed consistently to parent-report measures of both expressive and receptive language. Expansions predicted parent-reported expressive language skills. This study emphasises the need to capture what it is about parent language (i.e., discrete aspects) that influences child language acquisition and adds to the evidence that, alongside providing quality language input, the simple act of verbal imitation might be particularly important for autistic children’s language development.

### Relative Responsiveness and MLU

Relative parent responsiveness (i.e., responsive utterances divided by all parent utterances) only predicted later directly-assessed child receptive language skills; it was unrelated to the remaining five language outcomes. This finding adds to the ambiguity reported in other studies exploring summed responsive parent behaviours (both proportion and frequency) in relation to language skills in young autistic children (Choi et al., 2020; Flippin & Watson, 2015). Moreover, our findings suggest that whilst overall parent responsiveness plays a role in later language—in this instance understanding of language—by not exploring constituent parts of responsiveness, we are missing potential important associations, such as those discussed below.

Although we used parent MLU as a covariate here it should be noted that of all variables entered into regression models (including early child language), MLU remained the most consistent predictor (five of six) of child language outcomes in final regression models. Our findings support those of Choi et al. (2020) regarding the importance of parent MLU for later language abilities of autistic children. Our findings also emphasise that, in order to understand the relative importance of different aspects of the language learning environment more fully for autistic children, we must capture both *what* parents say to their young autistic children as well as *how* they say it.

### Imitations and Expansions

After controlling for earlier child words and parent MLU, we found that expansions of early language only predicted parent-report expressive communication in our sample, not parent-report vocabulary or directly-assessed receptive and expressive language. Findings both support and diverge from past literature. For example, parent expansions of child utterances *did* predict spoken vocabulary (measured the same way as in our study) in a small sample of minimally verbal autistic children (< 10 spontaneous words; McDuffie & Yoder, 2010). Parent MLU was not controlled for by McDuffie and Yoder (2010) which may contribute to discrepant findings—especially since we found it so consistently related to later child language here. Yet, Naigles (2013) *did* control for maternal MLU in their smaller, but more verbal sample, and found expansions positively related to subsequent receptive language scores. Expansions have likewise been strongly predictive of later language in non-autistic samples (Levickis et al., 2014; Taumoepeau, 2016).

Our sample was heterogenous language-wise, with baseline expressive MCDI vocabulary scores ranging from 0 to 630. Since past research has found divergent associations based on baseline language levels of children (Haebig et al., 2013), our equivocal findings may have been influenced by our language diverse sample. If we had the sample size to compare minimally verbal vs. more verbal children, for example, our patterns of results may have been clearer. Moreover, expansions were strongly correlated with our covariate child total words in the present sample (*r* = .798; note however that the assumption of multicollinearity for the regression analysis was not violated), which often carried significant unique predictive value at Step 2 in the regression models. This association is unsurprising since parents require child language to expand upon. However, it is plausible that this association limited our capacity to observe a unique statistical contribution of expansions to our outcomes. So, while it appears that expansions play a role in early language skills of autistic children, larger samples that permit sample stratification to identify contingent trends is required.

Parent imitations of verbal and non-verbal utterances predicted four of our child outcomes, namely parent-report expressive and receptive communication (i.e., per the VABS-II) and expressive and receptive vocabulary (i.e., per the MCDI). It was interesting that discrete responsive behaviours were predictive of parental report, but not directly assessed child language. Perhaps parent-report measures capture more nuance in what the child is capable of across different and familiar contexts over a short follow-up period in a way that direct assessments do not. Imitations of early words and sounds (measured in the same way as in the current study) have likewise been predictive of concurrent and subsequent language in non-autistic infants (Smith et al., 2018a, 2018b). Interestingly, however, McDuffie and Yoder (2010) found imitations of language were *not* uniquely predictive of spoken vocabulary (after controlling for child verbal utterances) in their sample of minimally verbal autistic children.

It was unclear whether McDuffie and Yoder (2010) measured imitations of *both* linguistic and pre-linguistic sounds. In our study, 19 children (38.8%) used < 10 spontaneous *words* in the parent–child interaction, but only three children (6.1%) had < 10 *utterances* of any type (i.e., whether comprising words or non-word vocalisations). Capturing whether a parent echoes linguistic *and* pre-linguistic sounds (as we did here) may be especially important in understanding the developmental trajectories of minimally verbal autistic children, who may struggle with communicative intent (i.e., when the child becomes aware of their role as a communicator able to send purposeful signals to a partner; Rowland & Fried-Oken, 2010). Imitations provide the child with immediate feedback that their verbal sounds have meaning to another person so may foster intentional communication (Bruner, 1975). Imitations likewise shape and strengthen early words by promptly reinforcing the adult language target (Tamis-LeMonda et al., 2001).

### Other Responsive Behaviours

It was surprising not to find any predictive value of responsive comments on child language given that this has been found by others, including in minimally verbal autistic children (Haebig et al., 2013; McDuffie & Yoder, 2010). Yet, there is inconsistency in the extant literature here. Whilst responsive comments were not predictive of later language for more verbally fluent (described as ≥ 5 words or phrases) young autistic children (Haebig et al., 2013), simple labelling has actually been found to be *negatively* associated with later language in a group of non-autistic, slow-to-talk toddlers (Levickis et al., 2014). We similarly found no associations between either type of responsive questions and later language outcomes. In non-autistic samples, parents’ use of responsive open-ended questions at 12 months *did not* predict later language (Smith et al., 2019), but when used at 24 months *they did* (Levickis et al., 2014); yet, simpler responsive yes/no questions used at 12 months were predictive of later language (Smith et al., 2019).

Regarding the aforementioned negative association between simple labelling and later language, Levickis et al. (2014) suggested that labelling did not provide the rich language input needed to promote language acquisition over the toddlerhood period. Similarly, different question types may likewise be more appropriate for specific periods of linguistic development. So, whilst responsiveness appears helpful for language acquisition, potentially specific behaviours still need to be tailored to the child’s individual level to effectively scaffold language learning (i.e., working in the Zone of Proximal Development; Vygotsky, 1962). Parents need to be aware of their child’s individual level of language proficiency to understand the input required to move them to the next linguistic level. Due to the heterogeneity of our sample, some autistic children may have benefitted from responsive commenting; others from the encouragement of child participation, choice-making and problem-solving afforded by question-asking (Taylor et al., 2009). What appears to be clear is that—for our heterogenous group of autistic children—parent responsive behaviours that explicitly met children at their language level (i.e., imitations and expansions), whatever their level, appeared important for all children.

## Limitations and Future Directions

Given our sample size, we were not able to explore the types of parent responsiveness which might be most critical for shaping early language acquisition in autistic children with more versus fewer baseline language abilities. Our sample size likewise precluded us from investigating whether findings differed depending on whether families were from Culturally and Linguistically Diverse (CaLD) versus non-CaLD backgrounds too. However, since most research, including autism research, is conducted with white, middle class cohorts (Rad et al., 2018), our diverse sample is a strength here. Further, our loss of data due to COVID-19 restrictions likely reduced our ability to detect differences in our analyses of directly-assessed child language. Finally, parent responsiveness was assessed based on a one-off, 10-min free play interaction between one parent and their child, using a standard set of materials. While these materials were selected to elicit a variety of interactions/play scenarios, and therefore capture a range of responsive behaviours, it is possible that capturing parent–child interactions across a variety of contexts might have yielded different results, especially when comparing more goal-orientated tasks (i.e., dressing, bath time, mealtimes) to free-play activities.

Moving forward, having standardised approaches to data collection in this field, for example creating a consistent approach to capture and assess parent responsiveness, could begin to address the challenges of comparing data across traditionally small samples in community-based research settings. Enhanced interoperability across data sets might address these limitations and our capacity to answer specific research questions. To explore generalisability of our findings, future research should also study different play scenarios, especially during more routine, naturalistic contexts across different cultural groups.

## Conclusions

Focusing on discrete behaviours may further our understanding of specific mechanisms underlying how autistic children acquire language in the context of everyday interactions with their regular social partners. Generalised responsiveness is encouraged as a way to promote broad social-communication skill acquisition in child-centered parent programmes for autistic children (i.e., Green et al., 2010) and infants at higher likelihood of developing autism (i.e., Whitehouse et al., 2019, 2021). Discrete parent responsive behaviours are also typically encouraged as a way to promote specific language development for autistic children (see Bruno et al., 2016 for more information). Our findings may be valuable for indicating the relative importance of different, teachable responsive behaviours that parents can readily incorporate within everyday routines to support language outcomes for their young autistic children, including the simple act of repeating back and building upon autistic children’s early words and sounds, whether or not these are yet used intentionally.
